# Factors Associated With Parent‐Reported Caries in Preschool Children From the KUNO‐Kids Health Study—Repeated Cross‐Sectional Analysis

**DOI:** 10.1111/cdoe.70078

**Published:** 2026-06-08

**Authors:** Áine M. Lennon, Katharina Fluch‐Maier, K.‐A. Hiller, Isabelle Schenke, Nils Gade, W. Buchalla, Susanne Brandstetter, Angela Köninger, Michael Melter, Christian Apfelbacher, Michael Kabesch, KUNO‐Kids Study group, Andreas Ambrosch, Petra A. Arndt, Andrea Baessler, Mark Berneburg, Romuald Brunner, Sara Fill Malfertheiner, André Franke, Stephan Gerling, Iris Heid, Stefanie Heinze, Wolfgang Högler, Sebastian Kerzel, Michael Koller, Michael Leitzmann, Áine Lennon, David Rothfuß, Martin Promm, Bianca Schaub, Stephan Weidinger, Sven Wellmann

**Affiliations:** ^1^ Department of Conservative Dentistry and Periodontology University Hospital Regensburg Regensburg Germany; ^2^ Department of Operative and Preventive Dentistry and Periodontology University of Göttingen Göttingen Germany; ^3^ University Children’s Hospital Regensburg (KUNO) Regensburg Germany; ^4^ Research and Development Campus Regensburg (WECARE), hospital St. Hedwig of the Order of St. John Regensburg Germany; ^5^ Clinic of Obstetrics and Gynecology St. Hedwig University of Regensburg Regensburg Germany; ^6^ Institute of Social Medicine and Health Systems Research Otto von Guericke University Magdeburg Germany

**Keywords:** child, dysbiosis, early childhood caries, family, habits and behaviours, paediatric dentistry, risk

## Abstract

**Objectives:**

This questionnaire‐based study aimed to assess parent‐reported caries (PRC) at ages 2–5 years and identify potential influencing factors using an exploratory, repeated cross‐sectional analysis within an ongoing birth‐cohort study.

**Methods:**

Parental factors included health, socioeconomic status, maternal age and dental check‐up attendance. Child factors included birth mode and weight, antibiotic, probiotic, bottle use, early dentition, weaning age, oral hygiene, diet, body mass index (BMI) and mouth‐breathing. Environmental factors included sibling caries, contact with other children, pets, or farms. Families (*n* = 1296) who completed the 2‐year caries questionnaire were included, corresponding numbers were 1276 at 3 years, 993 at 4 years, and 768 at 5 years. A subgroup comprised children who had visited a dentist since their last birthday (*n* = 735, at 2 years, *n* = 951, 891 and 662 at 3, 4 and 5 years respectively).

**Results:**

PRC rate was 0.4%, 1.3%, 2.7% and 4.4% for ages 2–5. Rates were 0.4%, 1.6%, 3.3% and 4.7% for the subgroup at the same ages. At Age 2, maternal migration background, lower paternal education and unemployment, late weaning, infrequent maternal dental check‐ups and pet contact were associated with PRC. Except for paternal education, the same factors remained associated in the subgroup. At Age 3, single‐parent status, maternal physical health, late weaning, sibling caries and pet contact were significantly associated with PRC. With the addition of infrequent maternal dental check‐ups, the same factors remained associated in the subgroup. At Age 4, early dentition, mouth‐breathing aged 1 were associated with PRC. Including late weaning, the same factors remained significantly associated in the subgroup. At Age 5, poor dietary and toothbrushing behaviour aged 2, younger mother, low BMI aged 1, infrequent maternal dental check‐ups, pet contact, and pacifier hygiene were associated with PRC. Except dietary behaviour aged 2, with the addition of paternal employment, the same factors were significantly associated in the subgroup.

**Conclusions:**

This study identifies key parental, child, and environmental risk factors for early childhood caries, revealing novel factors for further investigation and targeted prevention.

## 1 Introduction

Early childhood caries (ECC) remains a major public health concern worldwide and disproportionately affects socially disadvantaged children [1]. ECC significantly impairs children's quality of life and has lifelong consequences because it impacts important aspects of child development such as nutrition, growth, speech, sleep, and school performance [2].

ECC is defined as the presence of one or more decayed, missing or filled primary tooth surfaces in a child 71 months of age or younger [3]. ECC is classified as severe (S‐ECC) if there is smooth surface caries in a child under 3 years, or multiple affected teeth between the ages of 3 and 5 [4]. This disease, formerly known as nursing caries or baby bottle caries, is a severe, rapidly progressing form of dental caries that often develops soon after the primary teeth erupt. ECC has a unique clinical presentation because of the involvement of smooth surfaces, particularly the upper incisors, which are typically less affected in other forms of caries [5].

Dental caries is a biofilm‐driven disease, but the bacterial species found in ECC differ from those found in adult caries [6], or in healthy children [7, 8]. The oral microbiota acquired at birth undergoes shifts with the eruption of the primary teeth and evolves further in a healthy or dysbiotic direction during childhood depending on various factors.

While factors such as previous caries experience, diet, oral hygiene behaviours, and socioeconomic conditions are well‐established ECC risk factors [9, 10], many factors that potentially directly or indirectly shape the developing oral microbiome, including exposure to antibiotics, probiotics, and environmental contacts, have not been well investigated up until now [11, 12]. A portion of the population remains at risk of ECC despite these known associations, often requiring treatment under general anaesthetic [13, 14], highlighting the need to identify novel modifiable early life risk factors.

Because dental attendance in very early childhood is low, parent‐reported caries (PRC) represents a pragmatic way of capturing early disease in large population‐based studies, providing access to a group that might otherwise be missed due to limited clinical access [15]. However, since parents may not accurately recognise lesions themselves, PRC can underestimate true caries rates. Validation studies indicate low sensitivity (~9%–42%) but high specificity (~96%–97%) for parent‐reported caries [16]. Reporting is generally more reliable (sensitivity 50%, specificity 96%) if a child has recently visited a dentist and any caries present are likely to have been communicated to the parents [17].

Although several ECC risk indicators are well described, early life microbial and environmental influences remain insufficiently studied. Identifying these previously overlooked factors may offer new preventive approaches and help explain persistent inequalities in ECC prevalence.

The aims of this study were [1] to examine parent‐reported caries (PRC) over 4 years in preschool children using an exploratory, repeated cross‐sectional analysis within an ongoing birth‐cohort study and [2] to identify novel risk indicators for ECC with particular emphasis on microbial and environmental influences during critical developmental windows between birth and 2 years of age.

Within this framework, it was hypothesised that early life factors with the potential to alter microbiome development would be associated with higher PCR rates in one or more preschool years.

## 2 Materials and Methods

Findings are reported according to the Strengthening the Reporting of Observational Studies in Epidemiology (STROBE) checklist, see Data S1.

### 2.1 Participant Cohort

Newborns and their families (3196 in total) from Regensburg and rural surrounding areas in eastern Bavaria were recruited into the KUNO‐Kids health study between 2015 and 2020. The KUNO‐Kids health study is a prospective birth cohort designed to investigate child health and development from birth onward and includes comprehensive, medical, behavioural and socioeconomic data from the perinatal period. Each of the KUNO‐Kids questionnaires was developed by a multidisciplinary team and includes both validated instruments and study‐specific items tailored to the objectives of multiple research disciplines. The characteristics of this cohort have been described previously [18].

Baseline data were collected through standardised computer‐assisted personal interviews before hospital discharge. Follow‐up data were obtained by self‐administered paper questionnaires returned by post at 4 weeks, 6 months and at each subsequent birthday. Questionnaires were provided only in German. Underage mothers and those who were unable to fill out the questionnaire due to insufficient German language skills were not included in the cohort. Children who already had a sibling participating were also excluded, so that only one child per family took part. Families who responded to caries questions at ages 2, 3, 4 and 5 years were included in the present repeated cross‐sectional study. Families also reporting that their child visited a dentist in the past year were included in a subgroup. Participant numbers at each timepoint are given in Table 1. Families with missing caries data for a given year were excluded from that year's analysis, but included at other ages if data were available. All analyses were conducted as complete case analyses; no imputation was performed.

**TABLE 1:** Study population and subgroup composition with parent reported caries (PRC) rates by age.

Age	PRC	*n* (*n*)	Proportion subgroup %	PRC rate %
2	5 (3)	1295 (735)	56.8	0.4 (0.4)
3	17 (15)	1276 (951)	74.5	1.3 (1.6)
4	27 (27)	993 (891)	89.7	2.7 (3.3)
5	34 (31)	768 (662)	86.2	4.4 (4.7)

*Note:* Values for subgroup with dental visit always in parentheses.

### 2.2 Parent‐Reported Caries and Dental Visits

PRC was assessed annually from age 2–5 years, using a standard questionnaire item asking “has your child had one of the following complaints or diseases in the past year?” including caries (yes/no). In the same questionnaire, parents were asked “which of the following doctors have you consulted for your child in the past year?” including dentist (yes/no). These items and the response format were identical across all timepoints.

The study was approved by the Ethics Committee of the University of Regensburg (reference numbers: 14‐101‐0347 and 19‐1646‐101). Written informed consent was obtained from all participants aged 18 and over and from parents or legal guardians of minors.

### 2.3 Potential Influencing Factors

A wide range of potential influencing factors (see Table 2) was selected based both on previous ECC and S‐ECC research and factors with a possible influence on the developing oral microbiome in early childhood and thereby potentially contribute to dysbiosis and caries. Timing of data collection for each factor is shown in Table 2. Distribution of responses on potential influencing factors for study population and subgroup is given in Table 3.

**TABLE 2:** *p*‐values for significant or near significant associations between potential influencing factors and PRC. Values for subgroup in parentheses.

Factor	Question timepoint	Whole population (subgroup)				Direction
		Prc 2 years	Prc 3 years	Prc 4 years	Prc 5 years	
Socioeconomic factors						
Migration mother	Birth	**0.049 (0.003)**	n.s. (n.s.)	n.s. (n.s.)	n.s. (n.s.)	Non‐German citizenship 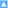 Risk
Single mother	Birth	n.s. (n.s.)	**0.001 (< 0.001)**	n.s. (n.s.)	n.s. (n.s.)	Single motherhood 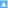 Risk
Paternal education	4 weeks	**0.041 (n.s.)**	n.s. (0.090)	n.s. (n.s.)	n.s. (n.s.)	Less education 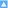 Risk
Paternal employment	1 year	**0.008 (< 0.001)**	n.s. (n.s.)	n.s. (n.s.)	0.054 (**0.039**)	Unemployment 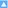 Risk
Child factors						
Birth mode	Birth	n.s. (n.s.)	n.s. (n.s.)	0.081 (0.090)	n.s. (n.s.)	Caesarean birth more often caries‐free
Birth weight	Birth	0.064 (n.s.)	n.s. (n.s.)	n.s. (n.s.)	n.s. (n.s.)	> 4 kg 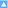 Risk
Baby bottle use in bed	6 months, 1 year	n.s. (0.08)	0.082 (n.s.)	0.078 (0.067)	n.s. (n.s.)	Bottle use 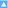 Risk
Early eruption of first teeth	6 months	n.s. (n.s.)	n.s. (n.s.)	**0.036 (0.029)**	n.s. (n.s.)	Early eruption 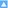 Risk
BMI 6 months	6 months	n.s. (n.s.)	n.s. (n.s.)	n.s. (n.s.)	n.s. (n.s.)	
BMI 1 year	1 year	n.s. (n.s.)	0.073 (n.s.)	n.s. (n.s.)	**0.015 (0.025)**	Low or high BMI 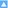 risk at 3 years, low BMI 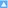 Risk at 5 years
BMI 2 years	2 years	n.s. (0.08)	n.s. (n.s.)	n.s. (n.s.)	n.s. (n.s.)	Low or high BMI 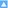 Risk
Antibiotic first 6 months of life	6 months	n.s. (n.s.)	n.s. (n.s.)	n.s. (n.s.)	n.s. (n.s.)	
Probiotics 1 year	1 year	n.s. (n.s.)	n.s. (n.s.)	n.s. (n.s.)	n.s. (n.s.)	
Probiotics 2 years	2 years	n.s. (n.s.)	n.s. (n.s.)	n.s. (n.s.)	n.s. (n.s.)	
Mouthbreathing 6 months	6 months	n.s. (n.s.)	n.s. (n.s.)	n.s. (n.s.)	n.s. (n.s.)	
Mouthbreathing 1 year	1 year	n.s. (n.s.)	n.s. (n.s.)	**0.039 (0.050)**	n.s. (n.s.)	Mouth breathing 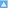 Risk
Mouthbreathing 2 years	2 years	n.s. (n.s.)	n.s. (n.s.)	n.s. (n.s.)	n.s. (n.s.)	
Toothbrushing score	2 years	n.s. (n.s.)	n.s. (n.s.)	n.s. (n.s.)	**0.006 (0.012)**	Lower score 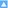 Risk
Nutritional score	2 years	n.s. (n.s.)	n.s. (n.s.)	n.s. (n.s.)	**0.031** (0.062)	Lower score 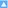 Risk
Oral thrush	1 year	n.s. (n.s.)	n.s. (n.s.)	n.s. (n.s.)	n.s. (n.s.)	
Weaning age	6 months, 1, 2, 3 years	**0.001 (< 0.001)**	**< 0.001 (< 0.001)**	0.058 **(0.043)**	0.093 (0.061)	Late weaning 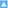 Risk
Parent factors						
Mother caries during pregnancy	Birth	n.s. (n.s.)	n.s. (n.s.)	n.s. (n.s.)	n.s. (n.s.)	
Dental complaints during pregnancy	Birth	n.s. (n.s.)	n.s. (n.s.)	n.s. (n.s.)	0.051 (0.088)	Complaints 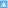 Risk
Mother age	Birth	n.s. (n.s.)	n.s. (n.s.)	n.s. (n.s.)	**0.001 (0.002)**	Younger mother 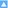 Risk
Maternal dental check‐up frequency	4 w postpartum	**0.005 (0.038)**	0.083 **(0.021)**	n.s. (n.s.)	**< 0.001 (< 0.001)**	< once a year 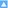 Risk
Pacifier hygiene	4 weeks	n.s. (n.s.)	n.s. (n.s.)	n.s. (n.s.)	**0.007 (0.032)**	Cleaning pacifier in mouth 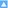 Risk
Mother caries 1st 6 months	6 months	n.s. (n.s.)	n.s. (n.s.)	n.s. (n.s.)	n.s. (n.s.)	
Mother PCS	1 year	n.s. (n.s.)	**0.007 (0.022)**	n.s. (n.s.)	n.s. (n.s.)	High PCS 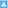 Risk
Mother MCS	1 year	n.s. (n.s.)	n.s. (n.s.)	n.s. (n.s.)	n.s. (n.s.)	
F‐SozU mother	1 year	n.s. (n.s.)	n.s. (n.s.)	0.074 (0.092)	n.s. (n.s.)	
Environmental factors						
Sibling caries	4 weeks	n.s. (n.s.)	**0.003 (0.001)**	n.s. (n.s.)	n.s. (n.s.)	Sibling caries 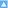 Risk
Child regularly spends time on farm	6 months	n.s. (n.s.)	n.s. (n.s.)	n.s. (n.s.)	n.s. (n.s.)	
Child contact to pets	6 months	**0.027** (0.082)	**0.002 (0.002)**	0.084 (n.s.)	**0.001 (0.002)**	Contact 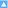 Risk
Child contact to other children 6 months	6 months	n.s. (n.s.)	n.s. (n.s.)	n.s. (n.s.)	n.s. (n.s.)	
Child contact to other children 1 year	1 year	0.073 (0.081)	n.s. (n.s.)	0.079 (0.067)	n.s. (0.081)	Less contact 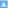 Risk
Child contact to other children 2 year	2 years	n.s. (n.s.)	n.s. (n.s.)	n.s. (n.s.)	n.s. (0.084)	Less contact 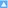 Risk

*Note:* Statistical significance is indicated by *p* ≤ 0.05; shown in bold, non‐significant results are marked “n.s”. Values, just outside the level of significance up to *p* = 0.095, are shown but not in bold to distinguish from significant results. 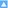 = increases risk.

Abbreviations: BMI, body mass index; F‐SozU, perceived social support; MCS, mental health‐related quality of life; PCS, physical health‐related quality of life; PRC, parent reported caries.

**TABLE 3:** Distribution of potential influencing factors by PRC age (2–5 years) in the study population and subgroup (dental visit). *n* (*n*): Number of participants in the total study population (subgroup with dental visit in parentheses); %(%) corresponding percentages for total population (subgroup in parentheses).

Factor	PRC Age 2		PRC Age 3		PRC Age 4		PRC Age 5	
	*n* (*n*) total	% (%)	*n* (*n*) total	% (%)	*n* (*n*) total	% (%)	*n* (*n*) total	% (%)
Child factors								
Birth mode: Caesarean section Yes	1295 (735)	31 (30)	1276 (951)	30 (29)	993 (819)	30 (29)	768 (662)	27 (27)
Birth mode: Caesarean section No		69 (70)		70 (71)		70 (71)		73 (73)
Birth weight < 2500 g	1287 (730)	5 (6)	1262 (940)	5 (5)	981 (810)	5 (5)	754 (648)	5 (5)
Birth weight > 2500 & ≤ 4000 g		85 (84)		85 (85)		85 (86)		86 (86)
Birth weight > 4000 g		10 (10)		10 (10)		10 (9)		9 (9)
Bottle use in bed Yes	1295 (735)	7 (7)	1276 (951)	7 (8)	993 (819)	7 (6)	768 (662)	6 (6)
Bottle use in bed No		93 (93)		93 (92)		93 (94)		94 (94)
Early eruption of first teeth Yes	1175 (679)	35 (37)	1152 (873)	36 (36)	876 (734)	36 (35)	683 (593)	38 (37)
Early eruption of first teeth No		65 (63)		64 (64)		64 (65)		62 (63)
BMI 6 months < 10th percentile	1161 (671)	10 (9)	1147 (868)	10 (10)	865 (726)	11 (10)	675 (586)	10 (10)
BMI 6 months 10–90th percentile		77 (78)		77 (76)		75 (77)		77 (77)
BMI 6 months > 90th percentile		13 (12)		13 (14)		14 (13)		13 (13)
BMI 1 year < 10th percentile	1074 (632)	18 (18)	1040 (778)	19 (18)	775 (651)	19 (19)	612 (531)	19 (19)
BMI 1 year 10–90th percentile		74 (75)		74 (73)		74 (75)		74 (74)
BMI 1 year > 90th percentile		8 (7)		7 (9)		7 (6)		7 (7)
BMI 2 year < 10th percentile	1077 (606)	11 (10)	812 (611)	10 (10)	594 (501)	11 (10)	443 (381)	10 (10)
BMI 2 year 10–90th percentile		81 (82)		82 (81)		81 (82)		83 (83)
BMI 2 year > 90th percentile		8 (8)		8 (9)		8 (8)		7 (7)
Antibiotic first 6 months of life Yes	1281 (723)	62 (62)	1261 (940)	62 (61)	981 (811)	63 (63)	759 (656)	61 (60)
Antibiotic first 6 months of life No		38 (38)		38 (39)		37 (37)		39 (40)
Probiotics 1 year Yes	1171 (680)	9 (9)	1134 (850)	9 (9)	852 (716)	9 (8)	666 (577)	9 (9)
Probiotics 1 year No		91 (91)		91 (91)		91 (92)		91 (91)
Probiotics 2 year Yes	1273 (721)	15 (15)	949 (706)	15 (14)	690 (577)	16 (15)	520 (447)	16 (16)
Probiotics 2 year No		85 (85)		85 (86)		84 (85)		84 (84)
Mouthbreathing 6 months Yes	1007 (567)	7 (7)	980 (741)	8 (8)	744 (629)	9 (9)	581 (505)	8 (9)
Mouthbreathing 6 months No		93 (93)		92 (92)		91 (91)		92 (91)
Mouthbreathing 1 year Yes	1007 (567)	11 (11)	967 (712)	10 (10)	724 (602)	11 (12)	562 (480)	11 (10)
Mouthbreathing 1 year No		89 (89)		90 (90)		89 (88)		89 (90)
Mouthbreathing 2 year Yes	1068 (594)	15 (15)	800 (596)	15 (15)	580 (486)	17 (17)	432 (367)	16 (16)
Mouthbreathing 2 year No		85 (85)		85 (85)		83 (83)		84 (84)
Toothbrushing score Low	1295 (735)	11 (8)	1276 (951)	32 (32)	993 (819)	36 (35)	768 (662)	39 (39)
Toothbrushing score Middle		42 (40)		32 (31)		31 (32)		29 (29)
Toothbrushing score High		47 (52)		36 (37)		33 (33)		32 (32)
Nutritional score Low	1295 (735)	11 (10)	1276 (951)	33 (32)	993 (819)	37 (36)	768 (662)	39 (40)
Nutritional score Middle		78 (80)		59 (60)		56 (57)		54 (53)
Nutritional score High		11 (10)		8 (8)		7 (7)		7 (7)
Oral Thrush Yes	1172 (676)	3 (3)	1132 (845)	2 (2)	851 (712)	3 (3)	660 (570)	3 (3)
Oral Thrush No		97 (97)		98 (98)		97 (97)		97 (97)
Weaning age < 6 months	811 (479)	32 (33)	1080 (811)	32 (32)	747 (623)	32 (32)	593 (515)	33 (33)
Weaning age 6–24 months		63 (62)		63 (64)		63 (63)		63 (63)
Weaning age > 24 months		5 (5)		5 (4)		5 (5)		4 (4)
Parent factors								
Mother caries during pregnancy Yes	1295 (735)	1 (1)	1276 (951)	1 (1)	993 (819)	2 (1)	768 (662)	2 (1)
Mother caries during pregnancy No		99 (99)		99 (99)		98 (99)		98 (99)
Mother dental complaints during pregnancy Yes	1278 (720)	35 (37)	1257 (936)	34 (34)	977 (807)	36 (35)	755 (652)	34 (34)
Mother dental complaints during pregnancy No		65 (63)		66 (66)		64 (65)		66 (66)
Migration mother Yes	1272 (720)	4 (3)	1254 (933)	4 (4)	975 (806)	4 (3)	755 (652)	4 (4)
Migration mother No		96 (97)		96 (96)		96 (97)		96 (96)
Mother age low	1280 (730)	10 (9)	1264 (944)	9 (9)	982 (812)	9 (9)	762 (656)	10 (10)
Mother age middle		79 (80)		80 (82)		80 (81)		79 (80)
Mother age high		11 (11)		11 (9)		11 (10)		11 (10)
Single mother Yes	1271 (719)	2 (2)	1252 (931)	2 (2)	974 (805)	2 (2)	754 (651)	2 (2)
Single mother No		98 (98)		98 (98)		98 (98)		98 (98)
Maternal dental check‐up frequency less than yearly	1162 (671)	7 (6)	1144 (855)	7 (6)	889 (739)	6 (5)	701 (605)	7 (6)
Maternal dental check‐up frequency at least yearly		93 (94)		93 (94)		94 (95)		93 (94)
Parent cleans dropped pacifier in own mouth Yes	1295 (735)	6 (6)	1276 (951)	6 (5)	993 (819)	5 (5)	768 (662)	5 (5)
Parent cleans dropped pacifier in own mouth No		94 (94)		94 (95)		95 (95)		95 (95)
Mother caries 1st 6 months Yes	1186 (686)	5 (5)	1163 (882)	5 (5)	883 (742)	5 (5)	690 (600)	5 (5)
Mother caries 1st 6 months No		95 (95)		95 (95)		95 (95)		95 (95)
Mother PCS Low	1149 (665)	15 (14)	1118 (835)	14 (14)	843 (706)	16 (16)	659 (566)	16 (15)
Mother PCS Middle		70 (70)		71 (71)		69 (69)		70 (70)
Mother PCS High		15 (16)		15 (15)		15 (15)		14 (15)
Mother MCS Low	1149 (665)	16 (15)	1118 (835)	15 (15)	843 (706)	15 (14)	659 (566)	14 (14)
Mother MCS Middle		70 (69)		71 (70)		70 (71)		70 (71)
Mother MCS High		14 (16)		14 (15)		15 (15)		16 (15)
F sozu mother Low	1175 (677)	20 (19)	1149 (861)	18 (17)	865 (723)	20 (18)	673 (579)	20 (19)
F sozu mother Middle		61 (61)		62 (63)		62 (63)		63 (63)
F sozu mother High		19 (20)		20 (20)		18 (19)		17 (18)
Education father > 10 years	1105 (642)	64 (64)	1046 (780)	64 (64)	799 (666)	64 (63)	634 (546)	66 (67)
Education father ≤ 10 years		36 (36)		36 (36)		36 (37)		34 (33)
Employment father Yes	1129 (653)	98 (98)	1105 (825)	98 (97)	835 (697)	98 (98)	647 (558)	98 (98)
Employment father No		2 (2)		2 (3)		2 (2)		2 (2)
Environmental factors								
Sibling caries Yes	1295 (735)	2 (2)	1276 (951)	2 (2)	993 (819)	2 (2)	768 (662)	1 (1)
Sibling caries No		98 (98)		98 (98)		98 (98)		99 (99)
Child regularly spends time on farm Yes	1191 (688)	8 (8)	1168 (884)	7 (8)	887 (744)	7 (7)	690 (600)	7 (7)
Child regularly spends time on farm No		92 (92)		93 (92)		93 (93)		93 (93)
Child contact to pets Yes	1188 (688)	50 (50)	1162 (882)	50 (50)	885 (744)	51 (53)	688 (598)	53 (53)
Child contact to pets No		50 (50)		50 (50)		49 (47)		47 (47)
Child contact to other children 6 months Yes	1178 (680)	55 (58)	1159 (879)	58 (59)	881 (739)	61 (62)	687 (597)	61 (60)
Child contact to other children 6 months No		45 (42)		42 (41)		39 (38)		39 (40)
Child contact to other children 1 year Yes	1168 (679)	75 (76)	1131 (846)	77 (77)	852 (713)	81 (81)	664 (574)	81 (82)
Child contact to other children 1 year No		25 (24)		23 (23)		19 (19)		19 (18)
Child contact to other children 2 year Yes	1290 (734)	90 (90)	961 (716)	90 (91)	702 (588)	91 (91)	524 (448)	93 (93)
Child contact to other children 2 year No		10 (10)		10 (9)		9 (9)		7 (7)

Abbreviations: BMI, body mass index; F‐SozU, perceived social support; MCS, mental health‐related quality of life; OHB, oral health behaviour; PCS, physical health‐related quality of life; PRC, parent reported caries.

Socioeconomic factors were: paternal employment status, paternal education, maternal migration background and single motherhood.

Child‐related factors were: birth mode, birth weight, BMI at 6 months, 1 and 2 years, baby bottle use in bed, early tooth eruption, antibiotic use in the first 6 months, probiotic use at 1 and 2 years, mouth‐breathing at 6 months, 1 and 2 years, nutritional behaviour, toothbrushing behaviour, oral thrush and weaning age.

Maternal factors included dental complaints during pregnancy, caries in the first 6 months postpartum, dental check‐up frequency, pacifier hygiene habits, physical and mental health‐related quality of life (HRQOL) measured using the mental or physical component scores (PCS or MCS) of the short form of the German Health Survey (SF‐12) and social support measured using the short form of the F‐SozU K‐14 survey.

Environmental factors were contact with other children at 6 months, 1 and 2 years, contact with pets, regular farm exposure, and having a sibling with caries.

For analyses involving these factors, complete case analysis was performed: only participants with complete data for all relevant variables were included. No imputation methods were applied.

### 2.4 Oral Health Behaviour (OHB) Scores

Nutritional and toothbrushing behaviour scores summarise multiple questionnaire items as previously described [19]. Scores reflect adherence to current oral health recommendations and were based on behaviours listed in Data S2. Maximum were 100 points for nutritional score (NS), 60 points for toothbrushing score (TS).

### 2.5 Categorisation of Interval Scale Data

Birth weight was divided into low (< 2500 g), medium (2500–4000 g) and high (> 4000 g). Weaning age was classified as early (< 6 months), normal (6–24 months) or late (> 24 months). Already having teeth at 6 months was defined as early tooth eruption. BMI at ages 6 months, 1 and 2 years of age was categorised as low (< 10th percentile), normal (10th to 90th percentile) or high (> 90th percentile) for girls and boys according to reference values from the U2 Untersuchungsheft (German neonatal health examination booklet) [20, 21]. Maternal age at time of birth was categorised as: younger (≤ 27 years), average 28–37 years and older (≥ 38 years). Mothers' attendance at dental check‐ups was separated into at least once yearly or less. Paternal education was classified as up to 10 years, 10 years, or more than 10 years school education. Maternal HRQOL was assessed using the SF‐12, a validated instrument comprising both physical and mental components [22, 23]. This generated mental (MCS) and physical component scores (PCS). These were divided into low (bottom 10%), moderate (middle 80%), and high (top 10%) categories. Maternal perceived social support was measured using the F‐SozU K‐14, a standardised validated short form social support questionnaire with 14 statements covering practical and emotional support, social integration, perceived social support and social strain [24]. Scores of 3.3 or less (bottom 10%) were categorised as low.

### 2.6 Conceptual Framework and DAG

In this study an ecological framework was adopted, in which factors influencing microbiome acquisition or subsequent modulation may alter the trajectory towards health or dysbiosis and therefore ECC [25]. Within this framework, parental socioeconomic characteristics are seen as upstream determinants, shaping the child's early home setting. Downstream exposures were grouped into three categories: *child factors* representing host biology and early behaviour, which may influence microbial colonisation (e.g., birth mode, mouth breathing, bottle use and toothbrushing behaviour), *parental factors* such as maternal caries at key timepoints and how pacifier hygiene is handled which may be vertical transmission pathways for cariogenic bacteria and *environmental factors*, representing possible microbial contact and horizontal transmission pathways external to the child (e.g., pets, farm visits, childcare settings) [26]. These exposures are thought to determine the development of dysbiosis ultimately leading to caries.

A directed acyclical graph (DAG) was constructed to illustrate the assumed causal relationship between these categories of early‐life factors and caries, see Figure 1. Dysbiosis is considered the mediator through which all exposures influence PRC.

**FIGURE 1:**
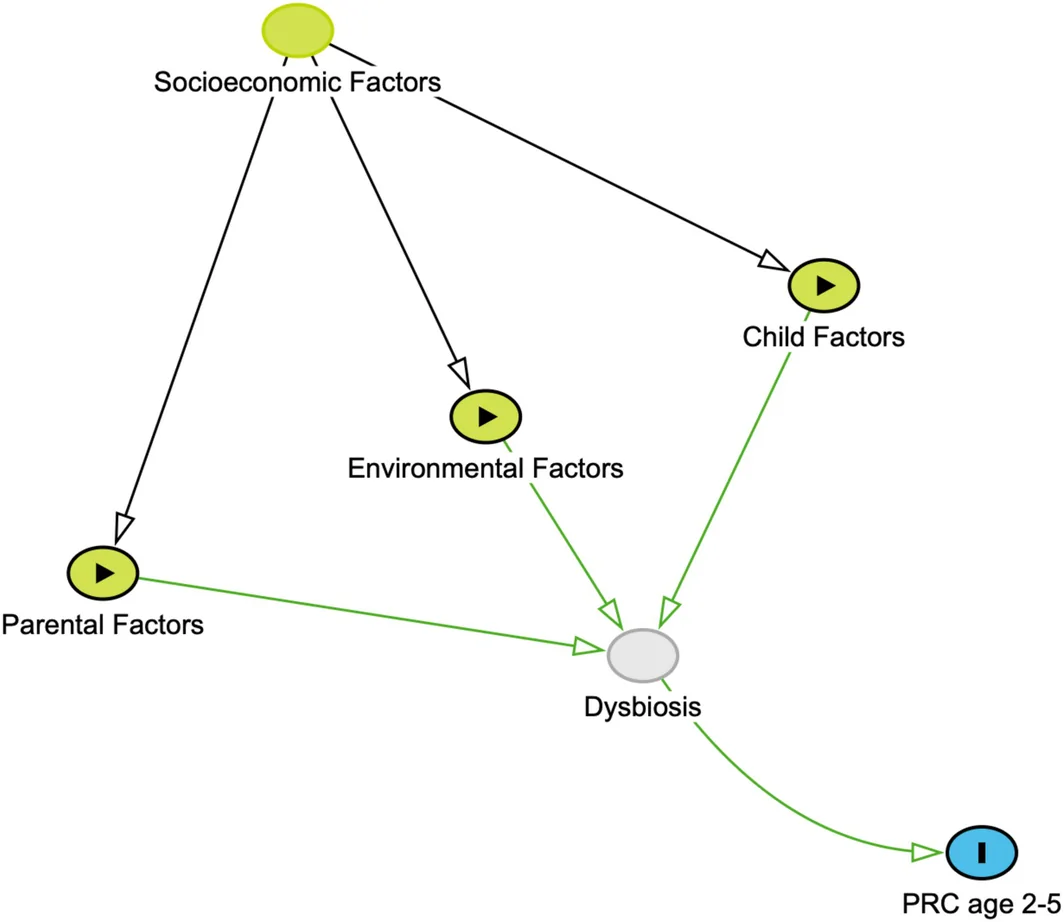
Simplified directed acyclical graph (DAG) showing the hypothesised pathways by which socioeconomic, parental, child and environmental factors may influence parent‐reported caries (PRC) through dysbiosis as the proximal biological mechanism. Nodes represent grouped domains rather than individual variables. Arrows reflect possible causal directions within the ecological microbiome focused framework. 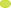 ancestor of exposure, 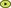 exposure, 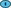 outcome, 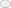 unobserved variable.

### 2.7 Data Analyses

Association between PRC at Ages 2, 3, 4 and 5 for the entire study population, and each potential influencing factor was tested using the chi‐squared test. The same testing was repeated for the subgroup who had visited a dentist in the past year. The level of significance was set at *α* = 0.05.

To explore potential confounding by upstream socioeconomic factors (migration, education, employment and single‐motherhood) selected log‐linear models were subsequently fitted. The SPSS log‐linear procedure models expected contingency‐table cell frequencies and is related to Poisson log‐linear modelling. However, because the very low outcome prevalence resulted in sparse contingency‐table cells, exponentiated coefficients/prevalence ratios are not reported. Only socioeconomic variables that were significantly associated with PRC at each age in univariate analyses were considered as potential confounders. Only exposures (variables from categories child, parent or environmental) showing significant associations with PRC at the same age were entered into these models.

For each age, exposures were entered into separate log‐linear models including PRC, and one potential confounder at a time. No models were run for Age 4 because no socioeconomic factors were significantly associated with PRC at this age. For Age 5, models were fitted only for the subgroup because no socioeconomic factors were associated with PRC in the main population at Age 5. Confounding was interpreted when the association between the exposure and PRC weakened or lost statistical significance after adjustment.

All statistical tests were carried out using SPSS for Windows, version 29, SPSS Inc., Chicago, USA. Figure 1. Directed acyclical graph was constructed using DAGitty V 3.1 [27].

## 3 Results

### 3.1 PRC

Non‐cumulative PRC rates, number of cases and number of respondents at each timepoint for the study population and the subgroup from Age 2 to 5 are presented in Table 1 (values for the subgroup are shown in parentheses). The analytical sample comprised 1295 participants at Age 2, 1276 (1.5% missing), 993 (23.3% missing), and 768 (40.7% missing) at Ages 3, 4, and 5 respectively, reflecting increasing attrition over time. Analyses were conducted as complete case analyses; therefore, sample size varied across variables due to missing data. The proportion of the study population who made up the subgroup is given for each age.

### 3.2 Factors Associated With PRC

Table 2 shows associations between all potential influencing factors tested, and PRC at ages 2, 3, 4 and 5 years for both the whole population and subgroup, including *p*‐values, direction of association and the timepoint at which each factor was recorded. All significant associations found showed higher PRC in the exposed group. Figure 2 presents −log_10_ transformed *p*‐values for each variable for each age and subgroup. This transformation produces a visual representation of the strength of each association, where higher bars indicate smaller *p*‐values and therefore stronger statistical associations. Only statistically significant associations, or those just outside the significance threshold, are shown.

**FIGURE 2:**
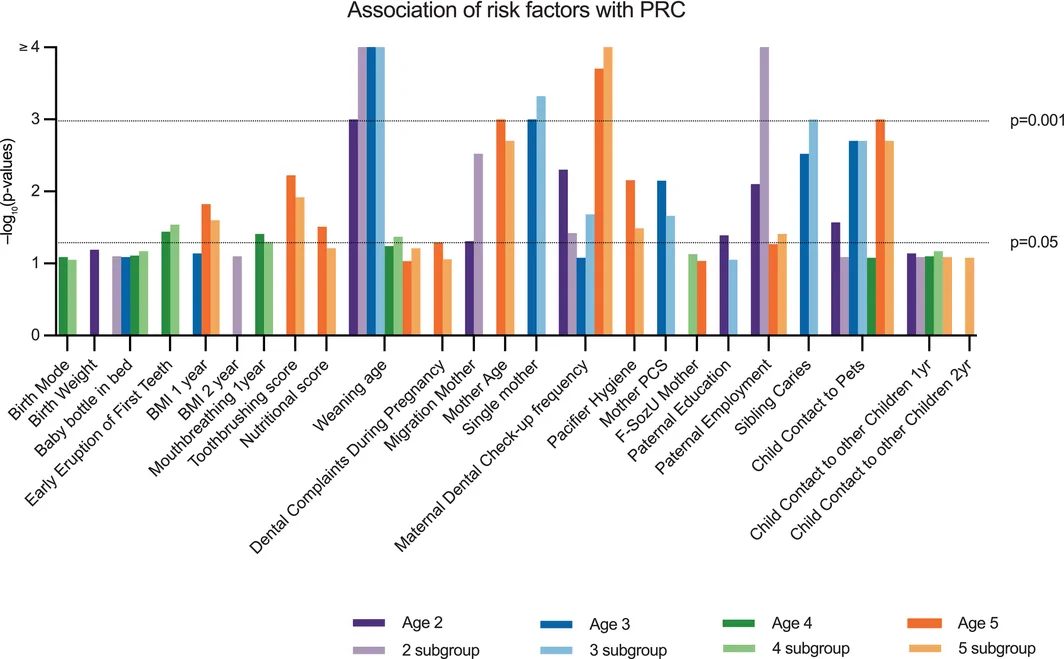
Negative log_10_ transformed *p*‐values for associations between parent‐reported caries (PRC) and variables with a statistically significant or near significant association across Ages 2–5 and corresponding subgroups. Bars represent the strength of statistical association, with higher values indicating smaller *p*‐values. Only associations with *p*‐values ≤ 0.095 are shown. Dashed lines indicate the threshold for significance: –log_10_(*p*) = 1.3 (*p* = 0.05) and –log_10_(*p*) = 3.0 (*p* = 0.001) for orientation. Subgroup refers to children who had visited a dentist within the year prior to the respective age. BMI, body mass index; F‐SozU, perceived social support; OHB, oral health behaviour; PCS, physical health‐related quality of life; PRC, parent reported caries; Yr, year.

### 3.3 Socioeconomic Factors

Each of the four socioeconomic factors was associated with PRC at least at one timepoint. Lower paternal education was significantly associated with PRC at Age 2 in the overall population but not in the subgroup. Paternal unemployment was significantly associated with PRC at Age 2 and in the subgroup at Ages 2 and 5. Single motherhood was highly significantly associated with PRC at Age 3 in both the overall population and the subgroup. Maternal migration background was significantly associated with PRC only at Age 2, with a highly significant association observed in the subgroup at this age.

### 3.4 Child‐Related Factors

Few perinatal characteristics were associated with PRC. No significant association was found for birth mode. Higher birth weight showed an association approaching significance at Age 2 but not later. No association was found for BMI at age 6 months, use of antibiotics, probiotics, or for oral thrush.

An association approaching significance was found for use of a baby bottle in bed at Age 2 in the subgroup, at Age 3 in the general population and at age 4 both in the general population and subgroup but not at Age 5. Early eruption of milk teeth was significantly associated with PRC at Age 4 in both the general population and in the subgroup, as was mouth breathing at Age 1.

Poor oral health behaviours at Age 2 showed significant association with PRC in the later preschool years. Low toothbrushing score was associated with PRC at Age 5 both in the overall population and in the subgroup. At Age 5, low nutritional score was associated with PRC and approached significance in the subgroup.

Duration of breastfeeding was one of the most consistently significant factors at all ages. Late weaning was highly significantly associated with PRC at Age 2 and at Age 3. At Age 4, early weaning was significantly associated with PRC in the subgroup and was just outside significance in the overall population. At Age 5, the association approached significance.

### 3.5 Parental Factors

No significant association with PRC was found for the following maternal factors at any age: caries during pregnancy, in the first 4 weeks or 6 months postpartum, perceived social support (F‐SozU score) or mental HRQOL (MCS score). Infrequent maternal dental check‐up attendance was associated with PRC, but only at Age 2.

### 3.6 Environmental Factors

Contact with pets showed one of the strongest environmental associations with PRC across all ages. At Ages 2 and 3, it was significantly associated, although the association was weaker in the subgroup. At Age 4, the association was weaker, and at Age 5, the association was again highly significant both in the overall population and the subgroup.

Less regular contact with other children at Age 1 was associated with PRC at Ages 2 and 4, but this was outside the level of significance. No significant association was found for contact with other children at 6 months or 2 years.

The association between having a sibling who had caries and PRC was highly significant but only at Age 3. On the other hand, no significant association was found for time spent on farm at any age.

### 3.7 Potential Confounding

Exploratory analyses using log‐linear models (Table 4) showed that most child, parent, and environmental PRC associations remained robust after adjustment for upstream socioeconomic factors. Migration and paternal unemployment both weakened the association between weaning age and PRC at Age 2. Paternal employment also showed confounding in the Age 5 subgroup for maternal age and checkup frequency, BMI Age 1, and pacifier hygiene. No confounding was found for pet contact at any age.

**TABLE 4:** Assessment of potential confounding by socioeconomic factors for significant associations with PRC.

PRC at age	Exposure	Exposure categories with PRC prevalence (*p* =)	Socioeconomic variable	Exposure categories with PRC prevalence	Interpretation
				Adjusted for socioeconomic variable	
				(*p* =)	
2	Weaning Age	≤ 6 months: 0.25%; 6–24 months: 0.12%; ≥ 24 months: 0.25% (0.031)	Migration	*Migration no*: weaning age ≤ 6 months: 0.25%; 6–24 months: 0.13%; ≥ 24 months: 0.13% *Migration yes*: weaning age ≤ 6 months: 0%; 6–24 months: 0%; ≥ 24 months: 0.13% (0.111)	Confounding
2	Weaning Age	≤ 6 months: 0.25%; 6–24 months: 0.12%; ≥ 24 months: 0.25% (0.031)	Education	*Education* ≤ 10 *years*: weaning age ≤ 6 months: = 0.28%; 6–24 months: 0.14%; ≥ 24 months: 0.14% (0.111). *Education* > 10 *years*: weaning age ≤ 6 months: = 0%; 6–24 months: 0%; ≥ 24 months: 0.14% (0.000)	No confounding
2	Weaning Age	≤ 6 months: 0.25%; 6–24 months: 0.12%; ≥ 24 months: 0.25% (0.031)	Employment	*Employment no*: weaning age ≤ 6 months: = 0%; 6–24 months: 0%; ≥ 24 months: 0.14% *Employment yes*: weaning age ≤ 6 months: = 0.27%; 6–24 months: 0.14%; ≥ 24 months: 0.14% (0.072)	Possible confounding
2	Maternal Dental Check‐up frequency	< once yearly = 0.17%; ≥ once yearly = 0.26% (0.040)	Migration	*Migration no*: check‐up < once yearly = 0.09% ≥ once yearly = 0.26% *Migration yes*: check‐up < once yearly = 0.09% ≥ once yearly = 0% (0.014)	No confounding
2	Maternal Dental Check‐up frequency	< once yearly = 0.17%; ≥ once yearly = 0.26% (0.040)	Education	*Education* ≤ 10 *years*: check‐up < once yearly = 0.09% ≥ once yearly = 0.28% *Education* > 10 *years*: check‐up < once yearly = 0.09% ≥ once yearly = 0% (0.018)	No confounding
2	Maternal Dental Check‐up frequency	< once yearly = 0.17%; ≥ once yearly = 0.26% (0.040)	Employment	*Employment no*: check‐up < once yearly = 0.10% ≥ once yearly = 0% *Employment yes*: check‐up < once yearly = 0.10% ≥ once yearly = 0.29% (0.033)	No confounding
2	Child Contact to Pets	Pet yes = 0.42%; Pet no = 0% (0.009)	Migration	*Migration no*: pet yes = 0.34%; pet no = 0% *Migration yes*: pet yes = 0.09%; pet no = 0% (0.005)	No confounding
2	Child Contact to Pets	Pet yes = 0.42%; Pet no = 0% (0.009)	Education	*Education* ≤ 10 *years*: pet yes = 0.39%; Pet no = 0% *Education* > 10 *years*: pet yes = 0.10%; Pet no = 0% (0.000)	No confounding
2	Child Contact to Pets	Pet yes = 0.42%; Pet no = 0% (0.009)	Employment	*Employment no*: pet yes = 0.38%; pet no = 0% *Employment yes*: pet yes = 0.09%; pet no = 0% (0.032)	No confounding
3	Weaning Age	≤ 6 months: 0.28%; 6–24 months: 0.65%; ≥ 24 months: 0.46% (0.003)	Single Mother	*Single Mother no*: weaning age ≤ 6 months: 0.28%; 6–24 months: 0.66%; ≥ 24 months: 0.38% *Single Mother yes*: weaning age ≤ 6 months: 0%; 6–24 months: 0%; ≥ 24 months: 0.09% (0.020)	Unlikely confounding
3	Mother PCS	PCS low = 0%, PCS middle = 0.72%, PCS high = 0.54% (0.009)	Single Mother	*Single Mother no*: PCS low = 0%, PCS middle = 0.73%, PCS high = 0.46% *Single Mother yes*: PCS low = 0%, PCS middle = 0%, PCS high = 0.09% (0.043)	Unlikely confounding
3	Sibling Caries	Sibling caries no = 1.18% sibling caries yes = 0.16% (0.042)	Single Mother	*Single Mother no*: sibling caries no = 1.12% Sibling caries yes = 0.08% *Single Mother yes*: sibling caries no = 0.08% Sibling caries yes = 0.08% (0.020)	No confounding
3	Child Contact to Pets	Pet yes = 1.03%; Pet no = 0.09% (0.001)	Single Mother	*Single Mother no*: pet yes = 0.88%; pet no = 0.09% *Single Mother yes*: pet yes = 0.18%; pet no = 0% (0.002)	No confounding
5 s	BMI 1 year	Underweight: 1.80%, norm: 2.45%, overweight 0.33% (0.029)	Employment	*Employment no*: underweight: 1.20%, norm: 2.40%, overweight 0.34% *Employment yes*: underweight: 0.17%, norm: 0.17%, overweight 0% (0.346)	Confounding
5 s	Toothbrushing score	Toothbrushing score low: 2.86%, middle: 0.91% high: 0.65% (0.006)	*Employment*	*Employment no*: TB score low: 0.31%, middle: 0% high: 0% *Employment yes*: TB score low: 2.01%, middle: 0.93% high: 0.62% (0.003)	No confounding
5 s	Mother Age	≤ 27: 1.31% (28–37): 2.76% > 37: 0.39% (0.005)	*Employment*	*Employment no*: age ≤ 27: 0.16% (28–37): 0.16% > 37: 0% *Employment yes*: age ≤ 27: 0.78% (28–37): 2.50% > 37: 0.31% (0.082)	Possible confounding
5 s	Maternal Dental Check‐up frequency	< once yearly = 1.0%; ≥ once yearly = 3.28% (0.003)	*Employment*	*Employment no*: check‐up < once yearly = 0%; check‐up ≥ once yearly = 0.33% *Employment yes*: check‐up <once yearly = 0.67%; check‐up ≥ once yearly = 2.66% (0.069)	Possible confounding
5 s	Pacifier Hygiene	Pacifier Hygiene yes: 0.65%; no 3.78% (0.026)	*Employment*	*Employment no*: pacifier hygiene yes: 0%; no 0.31% *Employment yes*: pacifier hygiene yes: 0.62%; no 2.94% (0.127)	Confounding
5 s	Child Contact to Pets	Pet yes = 3.49%; Pet no = 0.73% (0.001)	*Employment*	*Employment no*: pet yes = 0.33%; pet no = 0% *Employment yes*: pet yes = 2.50%; pet no = 0.83% (0.017)	No confounding

*Note:* Percentages indicate prevalence within each exposure category.

Abbreviations: 5 s, PRC age 5 subgroup; PCS, physical component score; PRC, parent reported caries; TB score, toothbrushing score.

## 4 Discussion

This study identified associations between several parent, child, and environmental factors and PRC in preschool children. Weaning later than 24 months and contact with pets were the factors most consistently associated with PRC across all ages. Associations below the statistical threshold such as those for birth mode, child probiotic use, or time spent on farms may reflect limited statistical power arising from low caries prevalence rather than true absence of effect. At Age 2 in particular, the small number of cases means that associations should be interpreted with caution. Given the known socioeconomic influence on ECC, selected multivariable analyses were undertaken to explore potential confounding where significant associations were found. Because PRC rates were very low, full multivariable models with multiple factors were not feasible. Very low prevalence produces unstable models which cannot meaningfully calculate odds ratios. For this reason, potential confounding was explored using separate 2‐ and 3‐variable log‐linear models.

This study used a repeated cross‐sectional design within a birth cohort, using annual questionnaires to assess PRC, due to the very low prevalence of caries in very early childhood. This design allowed us to analyse changes in PRC rates over time while considering a broad range of potential influencing factors and allowed us to use data from all available participants at each time point, ensuring a larger and more representative sample at each stage.

The inherent limitation of PRC, parental under or over reporting, must be kept in mind. While PRC cannot replace clinical data, it has been shown to correlate well with clinical findings for children over the age of 2 [28]. Although sensitivity can be low (up to 42%, increasing to 50% with a dental visit), specificity is high (96%) for parent or carer reported caries [16, 17]. PRC is a useful way to collect large‐scale data if a clinical examination is not feasible. Accordingly, parent‐reported caries has been successfully used as an outcome measure in previous studies investigating risk factors for childhood caries [29, 30].

Because of this concern, a subgroup with a dental visit was also investigated. Parents may not recognise a caries lesion themselves, but if the child has visited a dentist within the past year, they are likely to have been informed if caries was found. Although the proportion of children who had been to the dentist in the year running up to their second birthday was only 57%, the PRC rate was the same as in the study population. The size of the subgroup relative to the study population increased up to 90% Aged 4 and 86% Aged 5. At later ages, the PRC rate was slightly higher in the subgroup compared to the overall population, appearing to confirm a tendency for underreporting in this group.

Participation declined from 1295 at Age 2 to 768 at Age 5, consistent with typical attrition in birth cohorts [31, 32]. Another common problem in cohort studies is underrepresentation of disadvantaged groups, as they are lost earlier from long term studies [33]. The sociodemographic characteristics of this cohort have been described as above average so that the disadvantaged group at risk for ECC may be underrepresented [18]. Differential participation and reporting behaviour, such as social desirability bias, may contribute to underreporting of oral health problems in some groups [34] and should be kept in mind when interpreting these results.

Several factors were chosen because they are already established determinants of ECC such as socioeconomic status, maternal dental health and early child toothbrushing and nutritional behaviours [10, 35]. In addition, factors thought to influence the child's developing oral microbiome at important timepoints such as antibiotic and probiotic exposure were examined, such as contact with other children, farm and pet exposure, many of which have not been widely investigated in this context so far [36].

Weaning later than 24 months was the most consistently associated child‐based factor, across several time points, aligning with evidence that extended breastfeeding beyond 24 months increases risk for ECC [37, 38]. The association with early weaning was inconsistent; therefore, breastfeeding practices as a whole were not found to be consistently associated with PRC. The mechanisms involved are thought to be that prolonged frequent feeding, especially at night with lower salivary flow and delayed acid clearance, can favour cariogenic taxa [39]. The association between late weaning and PRC at Age 2 was found to be confounded by migration background and paternal employment, consistent with evidence that weaning age is influenced by socioeconomic and cultural factors [40].

Surprisingly, very little association was found between perinatal characteristics and PRC across the preschool years. Previous reports on mode of delivery on risk for ECC have shown conflicting results [41, 42]. An association was found between caesarean birth and lower PRC at Age 4. Higher birth weight (> 4 kg) showed a weak association at Age 2 confirming findings from previous studies [43, 44], but not at later ages.

Early eruption of first teeth, mouth breathing at Age 1, and low BMI at age 1 were associated with PRC in later preschool years, suggesting that exposures between 6 months and 1 year, when primary teeth erupt, the oral microbiome establishes, and early dietary and oral hygiene habits form, appear to contribute to subsequent caries, becoming evident at Ages 4 and 5 rather than immediately at Ages 2 or 3.

On the other hand, oral health behaviours such as nutritional score and toothbrushing score at Age 2, when the primary dentition is complete, showed a strong association with PRC at Age 5 confirming the link between poor early habits and ECC [45, 46].

Socioeconomic and parental factors were associated with PRC throughout the preschool years. Infrequent maternal dental check‐up and paternal unemployment were associated with PRC at early ages and again at Age 5. Other factors such as paternal education, maternal migration, or single mother status were associated with PRC at Ages 2 and 3 but not later, which may reflect decreasing participation of socioeconomically disadvantaged families in the study over time [18].

A notable observation was the consistent association between PRC and pet contact at all years investigated. This information was collected at 6 months, at the beginning of the window for deciduous tooth eruption and establishment of the tooth surface microbiome. Families were asked whether or not they had a pet, so regular rather than occasional contact can be inferred. Families have been shown to share some microbiota with their pets, so it is possible that children growing up in a household with a pet have different microbiota than those in a pet‐free household [47, 48]. Importantly, this association remained robust after adjustment for socioeconomic factors. Based on current knowledge, this association has not been previously reported and warrants further investigation as it is unclear whether this is due to (I) a direct biological effect related to the microbiome such as direct transfer of more cariogenic species from the pet to family members or (II) an indirect effect linked to behavioural differences between pet and pet‐free households. These might include more relaxed family habits regarding hygiene or less time available for oral hygiene care of children in families with pets compared to families without [49]. The other significant environmental factor was having a sibling with caries, which may be related to earlier transmission of cariogenic species or poor OHB within the family [50]. PRC was associated with less frequent contact with other children at Age 1 in childcare settings.

The strengths of this study include the inclusion of underexplored environmental factors, large sample size, and repeated data collection across the early childhood period within an established birth cohort. The use of annual questionnaires enabled detailed assessment of potential risk factors across multiple domains, collected from infancy up to preschool age.

### 4.1 Outlook and Limitations

This exploratory study supports the hypothesis that early life exposures with potential to influence oral microbiome development are associated with PRC in preschool children. However, because the findings represent cross‐sectional associations and cannot be interpreted as causal, confirmation in longitudinal studies is needed. The low prevalence of PRC, particularly at Age 2, limits statistical power and restricts the use of full multivariable models. Reliance on parent‐reported data may underestimate true caries rates. Future microbiome studies are needed to see if pet exposure alters the microbiome in a way that increases caries risk.

## 5 Conclusion

Within the limitations of the present study, PRC was associated with several early childhood factors consistent with an ecological microbiome centred framework. Factors plausibly influencing microbiome acquisition or development included late weaning, pet contact, sibling caries, child BMI, early tooth eruption, mouth breathing, oral hygiene, nutrition, and pacifier hygiene habits. Newly reported environmental factors, particularly pet contact, need to be further investigated before general preventive recommendations can be made.

## Author Contributions

Conceptualization: Á.M.L. Formal analysis: K.‐A.H., K.F.‐M., Á.M.L., N.G., I.S. Investigation: K.F.‐M., KUNO‐Kids study group. Resources: W.B., S.B., A.K., M.M., M.K. Statistical analyses: K.‐A.H. Data curation: K.‐A.H., K.F.‐M., Á.M.L., N.G., I.S., S.B. Writing – original draft preparation: Á.M.L. Writing – review and editing: Á.M.L., K.F.‐M., K.‐A.H., N.G., I.S., W.B., S.B., M.K., A.K., M.M., C.A. Funding acquisition: S.B., C.A., M.K. All authors have read and agreed to the published version of the manuscript.

## Funding

The KUNO‐Kids health study was funded by an EU research grant (HEALS: 603946). Further financial support was provided by the University Children’s Hospital Regensburg (KUNO‐Clinics) and the St Hedwig’s Hospital (Hospital Barmherzige Brüder Regensburg). The funders had no role in the design, data collection, data analysis, or reporting of this study.

## Conflicts of Interest

The authors declare no conflicts of interest.

## Supporting information


**Data S1:** Strobe checklist.

**Data S2:** Allocation of points towards Nutritional Score and Toothbrushing Score.

## Data Availability

The data that support the findings of this study are available on request from author KAH. The data are not publicly available due to privacy or ethical restrictions.
