# Supplementary material for: Factors Associated With Parent‐Reported Caries in Preschool Children From the KUNO‐Kids Health Study—Repeated Cross‐Sectional Analysis
Source: Community Dent Oral Epidemiol. 2026 Jun 8;54(5):596–612. doi: 10.1111/cdoe.70078 (PMC13669794; doi:10.1111/cdoe.70078)
Supplement: Supplementary file 1 [file CDOE-54-596-s001.docx]

| Nutritional score (max. 100 Points) | Points |
| --- | --- |
| Child did not have sugary drinks | **20** |
| Child had both sugary and sugar-free drinks | 10 |
| Child had only sugary drinks | 0 |
| Child drinks from a cup and only daytime | **20** |
| Child drinks from cup at night also | 10 |
| Child drinks from bottle during daytime | 3 |
| Child drinks from a bottle at night | 0 |
| Child does not eat sweets | **20** |
| Child eats sweets once a month | 18 |
| Child eats sweets several times a month | 15 |
| Child eats sweets once a week | 7 |
| Child eats sweets several times a week | 5 |
| Child eats sweets almost daily | 3 |
| Child eats sweets daily | 2 |
| Child eats sweets several times a day | 0 |
| Child eats fruit or vegetables several times a day | **20** |
| Child eats fruit or vegetables daily | 18 |
| Child eats fruit or vegetables almost daily | 15 |
| Child eats fruit or vegetables several times a week | 7 |
| Child eats fruit or vegetables once a week | 5 |
| Child eats fruit or vegetables several times a month | 3 |
| Child eats fruit or vegetables once a month | 2 |
| Child does not eat fruit or vegetables | 0 |
| Child avoided eating honey | 5 |
| Child avoided eating sugar | 5 |
| Child avoided eating sweets | 5 |
| These foods were avoided for dental health reasons | 5 |
| Tooth brushing score (max. 60 Points) | **Points** |
| Child’s teeth brushed after every meal | 20 |
| Child’s teeth brushed 2-3 times a day | 20 |
| Child’s teeth brushed once a day | 10 |
| Child’s teeth brushed once a week | 0 |
| Child’s teeth brushed occasionally | 0 |
| Child’s teeth not brushed | 0 |
| Parent brushes child’s teeth | **20** |
| Other member of household brushes child’s teeth | 20 |
| Child brushes own teeth under supervision | 10 |
| Child brushes own teeth | 0 |
| Children’s fluoridated toothpaste used | **20** |
| Adult fluoridated toothpaste used | 15 |
| Children’s non-fluoridated toothpaste used | 5 |
| Adult non-fluoridated toothpaste used | 5 |
| No toothpaste used | 0 |

**Supplemental Material 2.** Allocation of points towards Nutritional Score and Toothbrushing Score.
