# Supplementary material for: Factors Associated With Parent‐Reported Caries in Preschool Children From the KUNO‐Kids Health Study—Repeated Cross‐Sectional Analysis
Source: Community Dent Oral Epidemiol. 2026 Jun 8;54(5):596–612. doi: 10.1111/cdoe.70078 (PMC13669794; doi:10.1111/cdoe.70078)
Supplement: Supplementary file 2 [file CDOE-54-596-s002.doc]

STROBE Statement—checklist of items that should be included in reports of observational studies

*Page numbers refer to the submitted manuscript*

|  | Item No | Recommendation |
| --- | --- | --- |
| **Title and abstract** | 1 | (*a*) Indicate the study’s design with a commonly used term in the title or the abstract  Yes. Is on title page |
|  |  | (*b*) Provide in the abstract an informative and balanced summary of what was done and what was found  The abstract summarizes the aim, methods, results, and conclusions, highlighting the factors examined, the population studied, and the key findings. (page 2-3) |
| Introduction | | |
| Background/rationale | 2 | Explain the scientific background and rationale for the investigation being reported  Establishes the importance of ECC and S-ECC and the need for identification of novel risk factors (page 4-5) |
| Objectives | 3 | State specific objectives, including any prespecified hypotheses  Objectives are clearly stated at end of introduction. (page 5) |
| Methods | | |
| Study design | 4 | Present key elements of study design early in the paper  Can be found at start of methods. Repeated cross-sectional study within a birth cohort (page 5) |
| Setting | 5 | Describe the setting, locations, and relevant dates, including periods of recruitment, exposure, follow-up, and data collection  The setting is described as the KUNO-Kids health study, recruitment from the maternity department of the St. Hedwig clinic in Regensburg, Germany, and data collection between 2015 and 2020. Inclusion and exclusion criteria re explicitly stated (page 5) |
| Participants | 6 | (*a*) *Cohort study*—Give the eligibility criteria, and the sources and methods of selection of participants. Describe methods of follow-up  (Page 5)  *Case-control study*—Give the eligibility criteria, and the sources and methods of case ascertainment and control selection. Give the rationale for the choice of cases and controls  Not applicable.  *Cross-sectional study*—Give the eligibility criteria, and the sources and methods of selection of participants  Data for the outcome were collected for this study from participants who answered relevant oral health behaviour questions at the 2-year follow-up. (page 5 and 6) |
|  |  | (*b*) *Cohort study*—For matched studies, give matching criteria and number of exposed and unexposed  *Case-control study*—For matched studies, give matching criteria and the number of controls per case  Not applicable. |
| Variables | 7 | Clearly define all outcomes, exposures, predictors, potential confounders, and effect modifiers. Give diagnostic criteria, if applicable  Outcomes (Parent reported caries) and exposures (parental, child and environmental factors) are defined. (tables 2 and 4). Potential confounders such as higher socioeconomic status and increasing parental health literacy are addressed in the discussion. (page 12) |
| Data sources/ measurement | 8* | For each variable of interest, give sources of data and details of methods of assessment (measurement). Describe comparability of assessment methods if there is more than one group  Sources of data for outcomes and potential exposures are given, including individual questions from baseline interview and follow-up questionnaires up to 5 years of age. (pages 5-8 and table 2) |
| Bias | 9 | Describe any efforts to address potential sources of bias  Potential biases are discussed, such as selection bias and exclusion of non-German speakers and underage mothers, which may have an effect on the generalizability of the findings. (page 12-13) |
| Study size | 10 | Explain how the study size was arrived at  Included in the Methods on page 4: sample for the cohort (n = 3196) was derived from a fixed geographical catchment area and recruitment from Hedwig’s maternity hospital for a fixed time period. The sample for the present study (given in Table 1 and 4.) was determined by how many participants had responded to caries questions selected for in the 2–5-year questionnaires. Characteristics of the cohort has been previously published “Brandstetter S, Toncheva AA, Niggel J, Wolff C, Gran S, Seelbach-Göbel B, et al. KUNO-Kids birth cohort study: rationale, design, and cohort description. Mol Cell Pediatr. 2019 Jan 9;6:1.” |
| Quantitative variables | 11 | Explain how quantitative variables were handled in the analyses. If applicable, describe which groupings were chosen and why  Quantitative variables were categorized for analysis, with explanations provided for the chosen categories in materials section pages 7-8 “categorisation of interval scale data”. |
| Statistical methods | 12 | (*a*) Describe all statistical methods, including those used to control for confounding  Included in the Methods. (page 8) |
|  |  | (*b*) Describe any methods used to examine subgroups and interactions The selection of participants for the subgroup with dental visit is described on page 5 and 6. |
|  |  | (*c*) Explain how missing data were addressed  Missing data was avoided for the outcome, by including only those who had answered the caries question in each year’s questionnaire. A small degree of missing data was present for the potential influencing factors see table 4. |
|  |  | (*d*) *Cohort study*—If applicable, explain how loss to follow-up was addressed  only those with completed caries questions were included.  *Case-control study*—If applicable, explain how matching of cases and controls was addressed Not applicable.  *Cross-sectional study*—If applicable, describe analytical methods taking account of sampling strategy Included in the Methods. (pages 5 and 6) |
|  |  | (*e*) Describe any sensitivity analyses Not applicable. |

| Results | | |
| --- | --- | --- |
| Participants | 13* | Report numbers of individuals at each stage of study—eg numbers potentially eligible, examined for eligibility, confirmed eligible, included in the study, completing follow-up, and analysed included in tables 1 and 4 numbers for each potential exposure  See table 4. |
|  |  | (b) Give reasons for non-participation at each stage Reasons for non-participation in this cross sectional analysis were not investigated: Not applicable. |
|  |  | (c) Consider use of a flow diagram Not required. |
| Descriptive data | 14* | (a) Give characteristics of study participants (eg demographic, clinical, social) and information on exposures and potential confounders see Table 4 |
|  |  | (b) Indicate number of participants with missing data for each variable of interest see Table 4 in relation to total sample of 1296. |
|  |  | (c) *Cohort study*—Summarise follow-up time (eg, average and total amount)  Follow-up questionnaires were at 4wks, 6 months, 1 year and 2 years (Abstract and page 4) |
| Outcome data | 15* | *Cohort study*—Report numbers of outcome events or summary measures over time Not applicable |
|  |  | *Case-control study—*Report numbers in each exposure category, or summary measures of exposure  *See Table 3.* |
|  |  | *Cross-sectional study—*Report numbers of outcome events or summary measures distribution potential influencing factors for the study population and subgroup are given in Table 4. |
| Main results | 16 | (*a*) Give unadjusted estimates and, if applicable, confounder-adjusted estimates and their precision (eg, 95% confidence interval). Make clear which confounders were adjusted for and why they were included associations between parent reported caries and potential influencing factors, using p-values to indicate statistical significance. (Pages 8 to 11 and Table 2 and 4) |
|  |  | (*b*) Report category boundaries when continuous variables were categorized see Page 7-8 |
|  |  | (*c*) If relevant, consider translating estimates of relative risk into absolute risk for a meaningful time period Not applicable. |
| Other analyses | 17 | Report other analyses done—eg analyses of subgroups and interactions, and sensitivity analyses Subgroup analyses reported in results and Tables 2 and 4. |
| Discussion | | |
| Key results | 18 | Summarise key results with reference to study objectives see page 8 |
| Limitations | 19 | Discuss limitations of the study, taking into account sources of potential bias or imprecision. Discuss both direction and magnitude of any potential bias limitations (page 15) care in interpreting results in relation to general population page 12-13 |
| Interpretation | 20 | Give a cautious overall interpretation of results considering objectives, limitations, multiplicity of analyses, results from similar studies, and other relevant evidence  see discussion (pages 11-15) |
| Generalisability | 21 | Discuss the generalisability (external validity) of the study results  The manuscript discusses the generalisability of the findings, noting that the study population may not represent the most disadvantaged groups. (pages 12-13) |
| Other information | | |
| Funding | 22 | Give the source of funding and the role of the funders for the present study and, if applicable, for the original study on which the present article is based  Funding for present cross-sectional study-none  Funding for KUNOkids health study see funding statement (page 16) |
